# 基于低共熔溶剂的萃取分离技术及其应用研究进展

**DOI:** 10.3724/SP.J.1123.2020.07015

**Published:** 2021-02-08

**Authors:** Zexin ZHAO, Yinghe JI, Xiaomei LIU, Longshan ZHAO

**Affiliations:** 沈阳药科大学药学院, 辽宁 沈阳 110016; School of Pharmacy, Shenyang Pharmaceutical University, Shenyang 110016, China; 沈阳药科大学药学院, 辽宁 沈阳 110016; School of Pharmacy, Shenyang Pharmaceutical University, Shenyang 110016, China; 沈阳药科大学药学院, 辽宁 沈阳 110016; School of Pharmacy, Shenyang Pharmaceutical University, Shenyang 110016, China; 沈阳药科大学药学院, 辽宁 沈阳 110016; School of Pharmacy, Shenyang Pharmaceutical University, Shenyang 110016, China

**Keywords:** 低共熔溶剂, 分散液液微萃取, 固相萃取, 样品前处理, deep eutectic solvent (DES), dispersion liquid-liquid microextraction (DLLME), solid phase extraction (SPE), sample preparation

## Abstract

随着绿色化学的发展,开发和应用符合绿色化学要求的溶剂和方法备受关注。作为离子液体类似物,低共熔溶剂(deep eutectic solvent, DES)是通过氢键受体(hydrogen bond acceptor, HBA)和氢键供体(hydrogen bond donator, HBD)的氢键作用而形成的一种混合物,具有环境友好、制备简单、成本低、可生物降解等优点,在很多领域均有越来越广泛的应用。DES可以从不同样品中萃取和分离不同的目标化合物,其作为萃取溶剂具有独特的优势,可以获得较高的萃取效率且样品基质对分析过程的影响较小。在分散液液微萃取(dispersive liquid-liquid micro-extraction, DLLME)程序中,DES可以萃取复杂基质中的残留药物、金属离子和生物活性成分;与传统的萃取方法相比,该方法具有对有机试剂需求少,萃取效率更高等明显优势。而且,在DLLME中加入DES作为分散剂,能够加速萃取剂在样品溶液中的扩散,具有小型化、成本低等优点。相比于传统分散剂甲醇、乙腈的高挥发性、易燃性,DES的高稳定性、低毒性使其在绿色化学领域中更具有优势,应用更广。因此,DES与DLLME的结合近年来发展迅速。不仅如此,DES与固相萃取联合应用也具有广泛的应用前景,在与固相萃取小柱和搅拌棒联合应用时,DES可以作为洗脱剂,氢键供体及氢键给体的用量之比是洗脱效率的重要考察因素之一。在与磁性材料联用时,DES能与磁性多壁碳纳米管、磁性氧化石墨烯等纳米复合材料结合,通过氢键、*π-π*作用力和静电作用力等特异性吸附目标分析物。并且能够参与磁性凝胶和分子印迹聚合物的合成,推动磁性材料向绿色化学的方向发展,进一步拓展DES的应用。作为一类新兴的绿色溶剂,DES在化合物的萃取分离技术方面受到广泛关注,在不同的萃取技术中扮演了不同的角色,并表现出良好的性能,因此逐渐成为绿色化学领域的研究重点。该文整合了DES在萃取分离技术中的研究进展,介绍了DES的制备、性质和分类,对DES在DLLME和固相萃取中的应用进行了总结和归类,并展望了DES在萃取分离技术中的应用前景,为DES未来的应用提供参考。

## Abstract (en)

With the rapid development of green chemistry, the design and application of the related methods and requisite solvents have received increasing attention in recent years. Deep eutectic solvents (DESs) are mixtures formed from a hydrogen bond acceptor (HBA) and a hydrogen bond donor (HBD). Generally, ionic liquids (ILs) and DESs have similar physical and chemical properties, and hence, find application in the same fields. However, DESs have many advantages over ILs, such as non-toxicity, environmental friendliness, low cost, and biodegradability. Thus, there are many areas where DESs play a key role and act as new, efficient green extraction solvents. DESs can aid the extraction and separation of different target compounds from a variety of samples, thus promoting the rapid development of sample pretreatment technology. As extraction solvents, DESs offer unique advantages. In dispersive liquid-liquid microextraction (DLLME), DESs show incredible ability to extract residual drugs, metal ions, and bioactive components from complex matrices, which would require complicated sample preparation steps when using traditional organic extraction solvents. Compared with traditional organic extraction solvents, DESs have considerable merits of greenness, hypotoxicity, higher extraction efficiency, etc. Moreover, as a dispersant, a DES can accelerate the diffusion of the extractant in the sample solution during DLLME, owing to its benefits of miniaturization and low cost. Traditional dispersants such as methanol and acetonitrile have many disadvantages, including high volatility, flammability, and toxicity, while DESs are environmentally friendly. Therefore, the combination of DES and DLLME has recently gained prominence in the field of sample preparation. Additionally, the combination of DES and solid-phase extraction (SPE) has broad application prospects. By virtue of their diverse functions, DESs have been used as eluents, in combination with a solid-phase extraction column and a stir bar, to elute analytes from the sorbent surface. The molar ratio of the HBA and HBD is one of the important factors influencing the elution efficiency. DESs can be combined with magnetic multiwalled carbon nanotubes, magnetic graphene oxide, and other nanocomposites to specifically adsorb target analytes through hydrogen bonding, *π-π* forces, and electrostatic forces. In addition, the DES can be used in the synthesis of magnetic nanocomposites and molecularly imprinted polymers when combined with magnetic materials. Magnetic nanocomposites functionalized with DES show excellent performance and high efficiency in the extraction process. The combination of DES and magnetic materials would promote the development of magnetic materials for green chemistry and expand the application of DES to several other fields. However, to the best of our knowledge, research on the microstructure, physical and chemical properties, and extraction mechanism of DESs is still in its nascent stage. Therefore, exploring the theoretical mechanism and applications of new DESs with special functions would be an essential future research direction. This article integrates the research progress of DESs in extraction separation technology; introduces the preparation, properties, and classification of DESs; and summarizes the applications of DESs in DLLME and SPE.

有机溶剂常用于分析物的提取、分离、预浓缩等预处理技术中,然而它们的毒性、易挥发性和因此对环境造成的污染令人担忧。因此近年来,开发“绿色溶剂”是科学工作者们对绿色化学追寻的热点。离子液体因具有高度的热稳定性,极低的蒸气压,可变的黏度以及与有机和水性溶剂混溶等优点,曾被视为可作为有机试剂的替代品。然而一些报告表明,离子液体在环境中的积累可能对生态有害^[1]^,并且有一些研究表明离子液体生物相容性差,咪唑和吡啶类的离子液体甚至与有机试剂毒性相当^[2]^。因此开发一种低毒性,合成简单且具备一些离子液体性质的绿色溶剂具有十分重要的意义。2003年,由英国莱斯特大学Abbott教授提出的低共熔溶剂(DES)以其无毒且可生物降解、制备简单、成本低等性质而广受青睐^[3]^。DES是通过氢键受体和氢键供体通过氢键作用形成的一种混合物。由于分子间氢键的形成,DES的熔点比其任一组分的熔点都要低得多,因此在室温状态下常呈现液体状态^[4]^。由于组成DES的原料大多是天然存在的,因此DES符合绿色溶剂发展的特点。DES已经在化学合成、分离、纯化、电化学、能量储存等很多领域展现了它的优势^[5,6]^,甚至可以将其应用于色谱流动相中^[7]^。DES常分为疏水性DES和亲水性DES,最早由Abbott等提出的DES是由氯化胆碱-尿素合成的,具有较强的水溶性。随着对氢键受体(hydrogen bond acceptor, HBA)和氢键供体(hydrogen bond donator, HBD)的不断探索,亲水性DES的种类也越来越多。然而亲水性DES在水中易受到氢键的影响,不稳定,易分解,不能较好地应用于水溶液中的萃取分离,因此近年来对疏水性DES的研究也逐渐增多。因为其特殊的性质,DES在样品前处理的应用也越来越广泛,韩晓菲等^[8]^总结了这是由于以下4个特性引起的:(1)DES的凝固点可变,(2)密度高于水;(3)室温下黏度较高,(4)极性根据其组成而变换。Web of Science网站上的数据表明,从2015年至2020年6月,DES相关文章数量呈递增状态。其中将DES应用于萃取的文章共1348篇,同样呈现递增趋势(见图1)。这是因为DES的物理化学性质使其在萃取分离方法中成为挥发性有机溶剂和毒性有机溶剂的极佳替代品。随着分析技术的发展,DES广泛应用于残留药物、金属离子、生物活性物质等方面的萃取分离中。并且DES不再仅局限于分散液液微萃取法的应用,其在固相萃取与分散液液微萃取(dispersive liquid-liquid micro-extraction, DLLME)结合的技术中也得到了很好的应用。本文的目的在于整理整合DES在萃取分离过程中的应用及其研究进展,对DES的性质、在分散液液微萃取中的应用以及在与其他萃取分离手段联用中的进展进行了总结与归类,为DES未来的应用与发展提供参考。

**图1:**
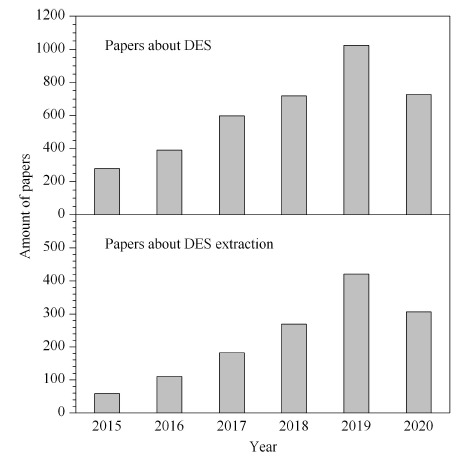
在Web of Science上搜索到的发表在2015年1月至2020年6月的相关文章数量

## 1 低共熔溶剂的制备、性质及分类

### 1.1 低共熔溶剂的制备

DES是氢键供体和氢键受体按照一定的物质的量之比,通过分子间氢键的合成而形成的一种绿色溶剂(见图2),典型的HBA为季铵盐等物质,典型的HBD包括多元醇,有机酸和糖等物质^[9,10,11,12]^。将固体原料结合产生了低共熔混合物,氢键或范德华力会干扰初始固体原料的结晶能力,故该混合物在环境温度下为液体,并表现出不同寻常的溶剂性质^[13,14]^。

**图2:**
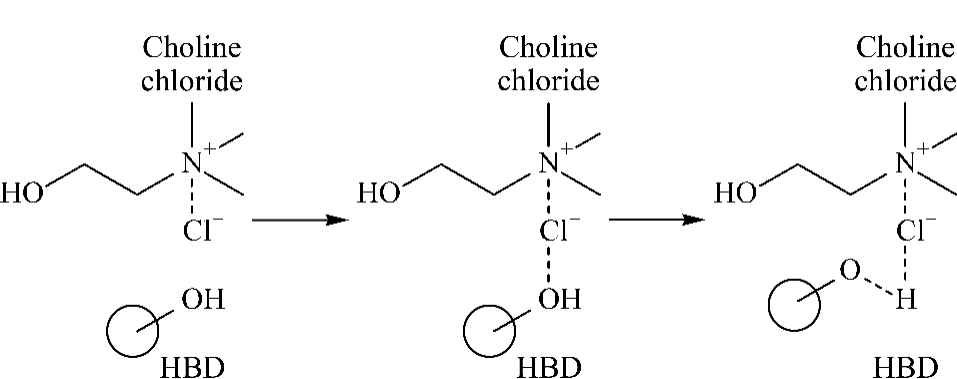
HBA (以氯化胆碱为例)和HBD作用示意图

DES的制备很简单,制备过程不会产生副产物,所以最终产物不需要纯化。除某些需要较高温度的组合物外,大多数DES可以通过在50~100 ℃下混合组分来制备^[4]^。2003年,Abbott等^[3]^通过在80 ℃下加热搅拌,使用氯化胆碱和尿素制备了第一个DES,该方法称为直接合成法,方法操作简单、得率高,因此被广泛用于DES的制备^[15]^。后来又有文献报道了合成DES的其他方法,例如,2009年,María等^[16]^通过将尿素水溶液与氯化胆碱水溶液混合,将得到的混合水溶液冷冻,然后通过冷冻干燥,获得了透明的黏稠液体,即DES。

### 1.2 低共熔溶剂的性质

1.2.1 熔点

DES的熔点是固体原料熔化形成DES时的温度,它决定了该DES适用温度的下限^[15]^。目前制备的多数DES熔点在70~150 ℃之间,其熔点比任何一个单一组分都低^[17]^。DES的熔点取决于HBA和HBD合成复合物的晶格能,氢键作用使HBA和HBD之间的电荷发生离域,导致混合物熔点降低,DES熔点降低程度与形成氢键的键能大小密切相关^[18]^。选择合适的HBA和HBD配比,可以得到该组分DES的最低熔点。

1.2.2 黏度

与离子液体类似,DES的黏度大多在0.01~5 Pa·s之间^[19]^。常温下,DES的黏度是水的几十至几百倍,主要原因是DES的网络结构使分子的流动性降低,导致黏度升高。DES的黏度受温度影响很大,随着温度的升高而急剧下降^[20]^。此外,其黏度还与组成及成分间的配比有关。

1.2.3 密度

大多数季铵盐与氢键供体合成的DES密度略大于1 g/cm^3^。DES的密度随着温度的升高而降低,如氯化胆碱-乙二醇(物质的量之比为1∶2)合成的DES在室温下密度为1.12 g/cm^3^,在353.15 K下密度为1.09 $g/c{m^{3}}^{}$ ^[21]^。

1.2.4 电导率

DES的电导率较高,比普通溶剂高几十甚至几百倍,这奠定了其广泛应用于电化学的基础^[18]^。DES的电导率受温度影响大,温度越高,DES电导率值越高,这是因为温度升高使DES黏度降低,离子运动变快,DES电导率值升高^[22]^。与此同时,DES结构中盐的质量比升高会导致DES电导率增大^[19]^。

### 1.3 低共熔溶剂的分类

Abbott等^[23]^将DES分为4个类型:Ⅰ类(无水金属卤化物和季铵盐), Ⅱ类(含结晶水的金属卤化物和季铵盐), Ⅲ类(氢键供体和季铵盐), Ⅳ类(金属卤化物和有机配体)。

第Ⅰ、Ⅱ类DES的合成依赖于卤化物中的阴离子和季铵盐通过共价键形成电荷被离域的新的阴离子。无水金属卤化物用来合成第Ⅰ类DES,其种类较少。相比之下,含结晶水的金属卤化物用来合成第Ⅱ类DES,其种类多且广泛存在,同时具有空气和水分不敏感性,可以用于第Ⅱ类DES的大规模生产过程中^[8]^。与前两类DES显著不同,第Ⅲ类DES的合成在于季铵盐中的卤素阴离子与氢键供体之间形成分子间氢键。这类DES具有易制备、成本低、可生物降解、与水不反应等优点^[24]^,而且氢键供体范围广泛,因此可以根据特定应用进行定制。第Ⅳ类DES形成原理是金属卤化物(MX*_n_*)经过不对称分裂成金属阴离子[MX*_n_*_-1_]^-^和金属阳离子[MX*_n_*_+1_]^+^,有机配体与金属阳离子络合,使阳离子电荷离域,电荷密度降低,降低阴离子和阳离子之间的库仑力,得到室温液体。

随着DES越来越受到关注,DES的类型并不局限于上述4类两种组分合成的DES。2005年,Imperato等^[25]^合成了基于多元醇-酰胺类-铵盐的三元DES。2011年,Choi等^[26]^和Dai等^[27]^提出天然低共熔溶剂(Natural deep eutectic solvent, NADES),随后又以氯化胆碱、甜菜碱、乳酸等天然物质为原料合成了一系列NADES,进一步扩展了DES的种类,使DES朝着更加绿色的方向发展。

## 2 DES在分散液液微萃取中的应用

分散液液微萃取是由Rezaee等^[28]^于2006年提出的微萃取技术。其特点在于萃取剂溶剂的细小液滴和分析物的接触,因此接触表面积高,从而加速了分析物的萃取。此外,因为该方法具有快速、萃取过程简单、成本较低、对样品以及有机溶剂需求量小的特点而被广泛应用^[29]^。实际上,DLLME由三组分溶剂系统组成:样品溶液、萃取溶剂和分散剂溶剂。DES由于其独特的溶剂特性优于萃取过程中使用的常规溶剂,因此DES和DLLME的结合近年来发展得十分迅速。DES-DLLME方法可以通过调整DES的组成,得到更好的萃取效率。根据DES-DLLME在残留药物萃取、金属离子的萃取、生物活性成分的应用做出了以下总结。

### 2.1 DES作为萃取剂

2.1.1 残留药物的萃取

食物和动物在生长成长过程中容易受到病原体和昆虫的侵害。农药和抗生素等药品在农业和畜牧业中广泛应用,以保护动植物,提高水果蔬菜以及动物源性食品的数量和质量。然而,在使用这些药物时,不适当的做法会导致食品中残留的药物水平超过极限,对人体健康造成危害^[30,31,32,33]^,因此,开发一种绿色、简便、灵敏的检测方法对食品安全有着重要意义。

Kachangoon等^[30]^提出了一种绿色、简便、灵敏的分析水、土壤和鸡蛋中新烟碱类杀虫剂残留量的方法,并结合高效液相色谱法(HPLC)分析。他们以癸酸为氢键供体,四丁基溴化铵为氢键受体,合成了一种疏水性的DES;在优化条件后,相对标准偏差(RSD)小于5.0%,重复性良好,检出限为0.001~0.003 μg/mL,与已有的杀虫剂分析方法相比,检测仪器简单但方法更加灵敏。疏水性DES的合成使得能够溶于水的药物得到更好的萃取,整个过程仅用了300 μL的DES及400 μL的乙腈分别作为萃取剂和分散剂,对环境的伤害小,环保且有效。

Jia等^[31]^建立了一种DES固化的泡腾片辅助微萃取技术结合高效液相色谱的方法,测定水、果汁、酒、醋样品中球果苷杀菌剂的残留量。作者以百里酚与辛酸合成的DES作为萃取剂,通过调节样品溶液的pH值来改变萃取剂的形式,进而达到提高萃取效率的效果。这种可以在不同pH下改变存在形式的萃取剂又被称为“可切换溶剂”。随着pH值的改变,可切换溶剂首先解离为离子形式,然后返回其分子形式,在亲水性和疏水性形式之间进行可逆变换,这导致溶剂和样品溶液之间表面积增大,萃取效率提高。同时,作者采用的泡腾片辅助微萃取法,是基于产生二氧化碳气泡的简单反应,用来加速萃取剂的分散。最后,作者采用悬浮有机液滴法(SFO)收集萃取剂。该方法省时高效,萃取溶剂与样品溶液达到平衡所需的时间仅为30 s,方法检出限为0.15~0.38 μg/L,回收率在77.4%~106.9%之间。与其他萃取方法相比,该法具有较低的检出限、较好的线性范围与回收率,可成功应用于实际样品中;同时,该方法有机溶剂消耗小,使用120 μL的DES作为萃取剂,更加绿色环保;采用pH调节DES的存在形式使DES可以更好地分散在样品溶液中;运用泡腾片辅助微萃取,萃取过程用时少;最后采用SFO方法收集萃取溶剂,降低了对仪器的要求,提高了分离速度。

Mokhtari等^[32]^合成了一种氯磷酸胆碱三组分DES,并应用于从蜂蜜样品中提取有机磷农药。该DES以磷酸胆碱氯化胆碱为氢键受体,二氯乙酸和癸酸为氢键供体。该方法首先将分析物从样品中提取到可溶于水的乙腈中。然后将萃取后的乙腈与制备的三元DES混合,DES作为萃取剂,乙腈作为分散剂,进一步净化样品。随后将收集到的DES进行气相色谱-质谱联用(GC-MS)分析。该方法经条件优化后,检出限为0.05~0.10 ng/mL,富集因子82~98,提取效率为82%~98%。与其他方法相比,该方法检出限较低、精密度较好,重复性好,可用于蜂蜜样品中目标分析物的提取和测定。三元DES的合成,令该方法萃取效率更高。

Jouyban等^[33]^采用一种在基质中形成DES,结合分散液液微萃取耦合固化有机液滴方法(DLLME-SFO)对牛奶样品中不同类别的农药进行提取和预浓缩。第一步,在牛奶基质中合成了亲水性DES,其在萃取过程中作为分散剂。第二步采用DLLME-SFO法,将前一步收集的有机相与疏水性的低共熔溶剂混合,对分析物进一步萃取。采用单因素优化法评估不同参数对方法效率的影响。DES比使用的大多数有机溶剂更安全、更环保。结果表明,该方法具有较低的检出限0.90~3.9 ng/mL,较高的富集因子320~445,较好的提取效率64%~89%。

使用DES作为萃取剂萃取残留药品,使萃取过程有机溶剂用量更小,方法更加绿色。尤其是将DES与其他分散液液微萃取技术耦合时,方法更加方便、高效。DES的应用,对于环境中残留药品的检测和净化更有意义。

2.1.2 金属离子的萃取

近年来,环境和食物中的金属元素污染对人类健康造成了一种威胁^[34]^。微量元素(例如铁、锌和钙等)在生物体代谢和生物合成过程中作为酶的辅助因子发挥着关键作用,被认为是人类生命生活中必不可少的,但是这些微量元素在高浓度的条件下,也会造成威胁^[35]^。重金属元素在人体中的积累达到一定水平也会导致严重甚至致命的疾病^[36]^。因此对环境和食物中金属离子的分析是十分必要的。

Menghwar等^[37]^将氯化胆碱和苯酚形成的DES溶剂用于富集橄榄油和水样中的铜离子。胆碱类化合物被公认为是低价且无害的有机盐,在实验过程中,通过调节氯化胆碱与苯酚的比例调节了实验的萃取效率。在优化的条件下,方法检出限为6.6 μg/L, RSD为4.0%~8.9%,实际样品中的回收率为92%~107%。方法的萃取步骤只需15 min就可以完成,与已有的DLLME方法相比较为快速。

Altunay等^[38]^以柠檬酸-蔗糖为原料,以3∶2的物质的量之比合成NADES,并采用超声辅助的方法,将DES用于萃取蜂蜜中的微量元素,最后用火焰原子吸收光谱仪进行测定。在最佳萃取条件下,该方法的检出限在0.077~0.29 μg/L内,富集因子在75~105内,RSD在1.3%~4.5%内,回收率在90.3%~98.4%之内。该方法与传统方法相比,富集因子较高、检出限较低。采用超声辅助的方法,加速了纳米粒微球的形成,提高了金属离子的回收和分离。并且其DES的合成步骤简单,价格低廉,是一种快速、简便、环境友好的技术。

从现有的化学药品制备的DES数量或类型是没有限制的^[39]^。Sorouraddin等^[40]^以薄荷醇、山梨醇和扁桃酸为原料合成三元DES,少量的DES同时用作络合剂和萃取溶剂萃取牛奶中的重金属离子(铬、铜、铅),萃取过程中几乎不使用任何有机溶剂。通过优化DES的组成与比例、样品体积、DES用量、分散剂种类和溶液pH等条件,得到方法定量限为0.38~0.42 μg/L, RSD在3.4%~5.2%之间。在本方法中,DES同时作为络合剂和萃取剂减少了有机溶剂的使用,简化了实验步骤,因而萃取过程高效、省时。

与其他萃取分离环境样品中金属离子的方法相比,DES成本低、合成简单且更加环保。部分DES溶剂可以同时作为络合剂与萃取剂,省去了络合剂的使用,生物可降解性高,可以作为环境废水净化的有效手段。

2.1.3 生物活性成分萃取

DES的应用使萃取效率大大提升,除了对残留药物、重金属的萃取分离外,DES还常用于有效成分的提取。然而比提高萃取效率更重要的是,获得的提取物保持其生物效应并且对人类无害,可作为食品和药物应用的可能和安全的替代品。Murador等^[41]^提出DES和离子液体萃取的生物活性化合物的生物利用度和生物效应优于传统有机溶剂。且DES毒性较低、不易挥发,在大多数情况下,可以在更短的时间内提高提取率。

Meng等^[42]^建立了一种使用氯化胆碱与1,2-丙二醇按1∶4的物质的量之比合成的DES,从蒲黄中提取4种生物活性类的黄酮。蒲黄花粉中的黄酮类成分具有广泛的极性和生物活性,有助于其药理活性和治疗效果。同时,该研究还建立了一种酸水解方法,使提取物中不易定量的黄酮苷转化为相应的糖苷配基形式,从而准确反映萃取效率。该方法在优化条件后,得到检出限为0.05~0.14 μg/mL,方法回收率在86.87%~98.89%之内,RSD < 4.38%。与用常规溶剂75%乙醇的提取效率相比,采用DES作为萃取溶剂效果更好。

Rathnasamy等^[43]^合成了一种功能性DES,结合微波辅助液-液分散微萃取,用于提取藻磷脂蛋白并测定了其生物活性。该方法利用甘油作为氢键供体,糖作为氢键受体,生成萃取溶剂DES用于提取藻蛋白,结合微波辅助萃取,利用响应面法对提取策略进行扩充,以从源处获得最大产物。

Hernández-Corroto等^[44]^利用DES从石榴皮中持续提取蛋白质和生物活性物质。因为石榴皮中含有较多的蛋白质、生物肽等物质,使用DES提取的蛋白质在水解产物中具有较强的降血压能力,通过HPLC-ESI-Q-TOF/MS鉴定为肽和多酚。该实验中共制备了8种DES,通过比较萃取效率,由于尿素是一种通常会干扰蛋白质测定的试剂,因此最终选择氯化胆碱作为氢键供体,乙酸作为氢键受体合成DES。与加速溶剂法作对比,在DES提取物的水解物中显示出更高含量的肽。

由上述文献可看出,DES可用于从不同样品中萃取分离不同种类的目标化合物,因此,DES作为萃取溶剂具有独特的优势,更多DES在药物残留、金属离子、生物活性成分等物质萃取分离中的应用见表1。

**表1:** DES作为萃取剂的应用

DES composition	Mole ratio	Matrix	Analytes	Detection technique	Ref.
Choline chloride/oxalic acid	1∶2	marine biological samples	Cu, Fe, Ni, Zn	ICP-OES	[45]
Choline chloride/oxalic acid	1∶2	biological fish samples	Cu, Fe, Zn	FAAS	[46]
Citric acid/xylitol; malic acid/ xylitol; citric acid/malic acid	1∶1	plant samples	As, Ca, Cd, Cu, Fe, K, Mg, Mn, Na, P, and Zn	ICP-MS, ICP-OES	[47]
L-Lactic acid/fructose/water	5∶1∶11	rhodiola rosea	phenyletanes, phenylpropanoids	HPLC	[48]
Choline chloride/polyalcohols, organic acids, sugar, urea	/	olive leaves	phenolic compounds	HPLC-DAD-ESI-TOF-MS	[49]
Choline chloride/glycerol	1∶1	byrsonima intermedia leaves	seven phenolic compounds	HPLC	[50]

ICP-OES: inductively coupled plasma-optical emission spectroscopy; FAAS: flame atomic absorption spectrometry; ICP-MS: inductively coupled plasma mass spectrometry; HPLC: high performance liquid chromatography; HPLC-DAD-ESI-TOF-MS: high performance liquid chromatography-diode array detection-electrospray ionization-time-of-flight mass spectrometer.

### 2.2 DES作为分散剂

在DLLME中,分散溶剂的唯一要求是:既能与样品溶液混溶,又能与提取溶剂混溶,以加速萃取溶剂在样品溶液中的分散,提高萃取效率^[51]^。目前分散剂大多使用的是乙腈、甲醇等,这些分散剂具有高挥发性、易燃性和相对毒性的缺点,因此在一定程度上限制了其在萃取分离领域中的应用。DES作为绿色低毒的分散溶剂,在该领域有着广阔的发展前景。

Shishov等^[52]^以四丁基溴化铵和甲酸合成的DES作为分散剂,1-辛醇作为萃取溶剂,结合注射器内流动系统和UV-Vis检测的自动化,测定饮料中铬(Ⅵ)的含量。合成的DES溶剂在注入系统后,因为有着色剂(络合剂)的存在,DES被分解,四丁基溴化铵对萃取起到盐析作用,甲酸为铬离子络合物的形成提供了合适的pH。与不加分散剂做对比,加入DES作为分散剂使萃取过程更快。其他离子对铬的吸附没有干扰,在最佳优化条件下,方法检出限为0.2 μg/L,重复性RSD小于8%,萃取效率为91%。DES作为萃取过程中的分散剂,加速了萃取剂在样品溶液的扩散。该方法具有自动化、小型化和成本效益等优点。

El-Deen等^[53]^以四丁基溴化铵和乙酸合成的DES为分散剂,2-十二烷醇为萃取剂,采用DLLME-SFO方法,萃取水样中的类固醇。与传统的分散剂甲醇、乙醇、丙酮对比,DES作为分散剂时提取效率更高。通过将响应曲面法和单因素法结合,对方法进行了优化。在最优条件下,该方法的检出限为1.0~9.7 ng/mL,预富集因子为44~112,日间日内精密度均小于5%。相对于其他萃取类固醇方法,该方法更加绿色环保,因此,DES可代替传统的有机分散剂用于样品前处理过程。

## 3 低共熔溶剂在固相萃取中的应用

### 3.1 与固相萃取小柱的联用

C_18_固相萃取小柱是一种操作简单快速的样品前处理装置,使用前先进行柱子的活化,然后加入样品,利用固体吸附剂将液体样品中的目标化合物吸附,接着淋洗,使目标化合物与样品中的干扰物分离,最后用洗脱液洗脱,以达到分离和富集目标分析物的目的^[54,55,56]^。因其具有操作过程简单快速,在很大程度上防止交叉污染等特性,固相萃取小柱在化合物的萃取分离中应用广泛且备受欢迎。

Li等^[57]^首先利用C_18_固相萃取小柱对山楂提取物中的咖啡酸进行了提取纯化,然后通过优化固相萃取过程,研究了不同用量之比的DES体系与甲醇混合作为洗脱剂对提取率的影响,得出当甲醇与甘油基DES在体积比3∶1时回收率较高,洗脱效果最好。结果表明,在与C_18_固相萃取小柱联用过程中,DES扮演了良好的洗脱剂的作用。与其他运用固相萃取小柱提取化合物的方法相比,其显著的优点为所用洗脱溶剂的低成本和低毒性。

### 3.2 与搅拌棒的联用

近年来,搅拌棒吸附萃取法逐渐引起人们的关注,搅拌棒吸附萃取作为一种集萃取、富集、净化为一体的新型样品前处理技术^[58]^,具有固定相体积大、溶剂用量少、富集倍数高、萃取容量高、环境友好、不需要外加搅拌子、可避免竞争性吸附、能在自身搅拌的同时实现萃取富集等优点^[59,60,61]^。广泛应用于食品、环境和生物样品分析的前处理过程中。

Negar等^[62]^开发了一种基于低共熔溶剂的新型搅拌棒微萃取技术,用于从蔬菜和果汁样品中进行槲皮素和桑色素的预浓缩和提取。首先将目标分析物从水溶液样品中萃取到1-十一烷醇中,然后进一步反萃取至亲水性DES和甲醇的混合物中。氯化四甲基铵/乙二醇的亲水性DES体系作为理想的DES组成,以搅拌速度1000 r/min,萃取时间40 min,完成对样品中槲皮素和桑色素的萃取。实验结果表明,相比于传统溶剂,DES绿色、高效的特点在萃取分离中体现出了重要的作用。Mahboob等^[63]^首次将DES同时作为洗脱剂、分散剂和萃取剂。首先,通过搅拌棒吸附法将分析物吸附在搅拌棒的表面,接着,用所合成的DES (氯化胆碱-乙二醇)对分析物进行洗脱,该DES在后面的漂浮固化有机液滴-分散液液微萃取过程中也发挥着分散剂的作用。然后,将洗脱液与衍生化试剂和DES (氯化胆碱-正丁酸,作为萃取剂)混合,将混合物注入去离子水中,置于冰浴中使萃取剂液滴固化。该方法回收率较高,且富集因子可达2999,表明了DES功能的多样性。Mohammad等^[64]^将搅拌棒吸附萃取法与基于低共熔溶剂的分散液液微萃取法结合起来,萃取率达到92%,在气相色谱-质谱分析之前,成功衍生和萃取了某些酸性农药。这些报道极大地拓展了DES的研究思路。

### 3.3 与纳米材料的联用

3.3.1 与分散材料的联用

分散材料种类繁多,其中,在化合物的萃取分离领域,磁性多壁碳纳米管、碳纳米角、碳纳米纤维、磁性氧化石墨烯及其功能化后的纳米复合材料得到了广泛的应用。上述材料均具有独特的结构及优良的吸附性能,通过氢键、*π-π*和静电等作用力特异性吸附目标分析物,在固相萃取过程中发挥着至关重要的作用^[65,66,67,68,69]^。DES种类多样,通过化学键连接或超声包覆等方式修饰在纳米材料表面,可以合成并筛选出对目标分析物有特异性吸附的吸附材料。基于DES功能化后的磁性纳米复合材料在从某些基质中萃取目标化合物的过程中表现出了更好的性能及更高的萃取效率。

Zhao等^[70]^采用超声辅助的磁固相萃取测定果蔬中的农药残留。在样品预处理过程中,先用DES萃取分离目标分析物,再用功能化的磁性多壁碳纳米管对样品溶液进行净化,选择脯氨酸/丙二醇以物质的量之比1∶3为最佳DES体系,萃取效率达98.0%。当代农药的不合理使用导致农产品中某些农药含量超标,农药残留也是造成环境污染的主要因素之一,运用该方法可高效地实现农药与果蔬样品溶液的萃取与分离,为检验农产品中农药含量提供思路,极具参考价值。Zhao等^[71]^合成了一种基于低共熔溶剂的磁性凝胶,将这种绿色新型的材料用于水性护肤品中性激素的提取与分离,进一步拓展了DES的应用领域。Li等^[72]^在磁性氧化石墨烯的表面上制备了新型分子印迹壳聚糖微球,使用DES作为功能单体和模板,萃取富集水中的氯酚,在温度40 ℃,萃取时间35 min时萃取量最高,为86.90 mg/g。与传统材料相比,其具有较高的选择性和提取能力。Mohamed等^[73]^利用烯丙基三苯基溴化的DES-官能化碳纳米管去除水中的汞元素,发现在pH为5.5,吸附量为5 mg,接触时间28 min时,水中的有害元素汞可以被有效去除。结合之前的文献,单独的DES可以对金属离子进行吸附,实现净化功能,而将亲水性DES与纳米材料结合后,其在水溶液中的良好分散作用被体现出来,使得目标分析物能更高效快速地被吸附在纳米材料上。因此,联用技术操作简便、省时省力的特点在重金属去除领域有着广阔的应用,DES在其中发挥了较大的作用。

3.3.2 与分子印迹聚合物材料的联用

分子印迹聚合物(molecular imprinted polymers, MIPs)是能够对特定的目标分子及结构类似物特异性识别和分离的高分子聚合物,它不仅对目标分子具有与生物抗体类似的高选择性,而且具有较好的化学稳定性和机械强度,因此广泛应用于食品、环境及药物分析等领域^[74,75,76,77]^。MIPs的优异性很大程度上依赖于一些有机溶剂的使用,如正己烷、甲苯、甲醇、丙酮等,从而使整体柱的孔隙度及其形貌更符合分析要求。然而,大量使用具有挥发性的有机溶剂会造成环境污染,与发展绿色化学的宗旨相背离。当前,越来越多关于MIPs的研究报告集中于开发新型绿色的合成方法。

Jin等^[78]^设计合成了一种基于低共熔溶剂的离子介导MIPs整体柱,对绿原酸的保留行为进行了研究。发现当致孔剂中DES的含量较大时,谱图拖尾严重,而在DES取用量为1200 mL,模板分子、功能单体、交联剂物质的量的比为1∶4∶20时,整体柱保留性能最佳,为绿原酸印迹聚合物的绿色合成提供了新的思路。Ge等^[79]^合成了一种杂化的磁性介孔分子印迹聚合物作为一种新型的磁性固相萃取吸附剂,用于从大鼠尿液样本中选择性识别马兜铃酸Ⅰ和Ⅱ。将DES体系胆碱氯化物氯化胆碱/乙二醇用作洗脱液,方法廉价环保,马兜铃酸的回收率在86.7%~94.3%之间。该方法将分子印迹聚合物与DES共同运用到目标分析物的萃取过程中来,磁性介孔分子印迹聚合物作为吸附剂,DES作为洗脱剂,二者联合后,相比其他处理生物样品的方法,该方法避免了使用传统有机溶剂作为洗脱剂,对环境友好且操作简便,准确度高,为检测生物样品中的马兜铃酸Ⅰ和Ⅱ开发了一种很有前途的方法。

## 4 结论与展望

作为一类新型的绿色溶剂,低共熔溶剂在化合物萃取分离领域的应用受到广泛关注,在不同的萃取方法如固相萃取和液液分散微萃取中,分别扮演了不同的角色,发挥了不同的作用,逐渐成为绿色化学化工领域研究的重点。其良好的溶解性、热稳定性、可生物降解性及合成过程的简便性,成为低共熔溶剂相比于其他萃取剂的重要优势。纵观文献,DES在生物活性成分、金属离子、有机物的萃取分离方面仍然处于不断探索的阶段,还有许多问题需要深入探究。当前对低共熔溶剂的研究大多集中在新型低共熔溶剂的设计与开发以及对新的应用领域的探索上,着重设计并合成了环境友好及生物相容性较好的新型DES。然而,对于DES的理论机制、物质之间的微观结构分析及萃取分离机理等方面的研究较为缺乏。物质的理化性质是其应用的基础,因此,着重探索低共熔溶剂潜在的理论机制,开拓具有特殊功能的新型低共熔溶剂的应用空间,成为之后工作当中的重要研究方向。此外,借助于其他萃取技术,DES可以发挥更好的萃取分离效果,同时,其他技术在DES的辅助下,萃取效率成倍提高,能够达到一加一大于二的效果。因此,应继续探索DES与其他技术联合使用的研究道路,促进DES的萃取分离技术深入发展。
